# Multidrug-Resistant *Escherichia coli* Accumulated by Freshwater Bivalves: An Underestimated Risk for Public Health?

**DOI:** 10.3390/pathogens13080617

**Published:** 2024-07-25

**Authors:** Joana C. L. Martins, Ana Gonçalves, Conceição Fernandes, Edna Cabecinha, Sandra Monteiro, Hugo Guedes, Gonçalo Almeida, Juliana Garcia, Gabriela J. da Silva, Simone Varandas, Maria J. Saavedra

**Affiliations:** 1 AB2Unit—Antimicrobials, Biocides & Biofilms Unit, Veterinary Sciences Department, University of Trás-os-Montes and Alto Douro (UTAD), 5001-801 Vila Real, Portugal; joanaalourenco@gmail.com (J.C.L.M.); ana-isabel1997@hotmail.com (A.G.); conceicao.fernandes@ipb.pt (C.F.); edna@utad.pt (E.C.); smonteir@utad.pt (S.M.); simonev@utad.pt (S.V.); 2 CITAB—Centre for the Research and Technology of Agro-Environment and Biological Sciences, Institute for Innovation, Capacity Building and Sustainability of Agri-Food Production (Inov4Agro), University of Trás-os-Montes e Alto Douro, 5001-801 Vila Real, Portugal; juliana.garcia@aquavalor.pt; 3 CECAV—Veterinary and Animal Research Centre, Associate Laboratory for Animal and Veterinary Science (AL4AnimalS), University of Trás-os-Montes and Alto Douro, 5000-801 Vila Real, Portugal; 4 AquaValor—Centro de Valorização e Transferência de Tecnologia da Água, 5400-342 Chaves, Portugal; 5 CIMO—Centro de Investigação de Montanha, SusTEC-Laboratório Associado para a Sustentabilidade e Tecnologia em Regiões de Montanha, IPB—Instituto Politécnico de Bragança, Campus de Santa Apolónia, 5300-253 Bragança, Portugal; 6 CIBIO—Centro de Investigação em Biodiversidade e Recursos Genéticos, InBIO Laboratório Associado, Universidade do Porto, Campus de Vairão, 4485-661Vairão, Portugal; 7 National Institute for Agricultural and Veterinary Research I.P. (INIAV), Lugar da Madalena, 4485-655 Vila do Conde, Portugal; hugo.guedes@iniav.pt (H.G.); goncalo.almeida@iniav.pt (G.A.); 8 Centre for Study in Animal Science (CECA-ICETA), Associate Laboratory for Animal and Veterinary Science (AL4AnimalS), University of Porto, 4099-002 Porto, Portugal; 9 Faculty of Pharmacy, Center for Neurosciences and Cell Biology, University of Coimbra, 3000-548 Coimbra, Portugal; gjsilva@ci.uc.pt

**Keywords:** *Anodonta anatina*, antimicrobial resistance, one health, phylogenetic, animal health

## Abstract

As bioindicators, freshwater bivalves are crucial for the assessment of the contamination impact on different levels of biological integration. *Escherichia coli* is used as a bioindicator of water fecal contamination, representing a critical global concern, especially with the rise of multidrug-resistant (MDR) strains. Phylogenetic diversity, pathotypic characterization, and antibiotic resistance profiles of *E. coli* isolated from freshwater bivalves (*Anodonta anatina*) were assessed. Samples were collected from the Tua River in Northern Portugal, from two different sites, Chelas and Barcel, representing different degrees of contamination. Antimicrobial susceptibility testing was performed by the disk diffusion method, and characterizations of the phylogenetic groups and pathotypes were assessed by PCR-multiplex and real-time PCR-multiplex, respectively. Results showed that 60% of isolates were characterized as MDR, including resistance to carbapenems, considered the last resort against multidrug-resistant bacteria. Within this study, it was also possible to verify the antimicrobial resistance (AMR) profile differences between the two sampling sites, with bivalve isolates from the Chelas site showing a higher percentage of antibiotic resistance. Among the *E. coli* isolates, the highest prevalence (55%) was observed in group B1, followed by group D or E (15%), group A (10%), and group E or Clade I (10%). None of the isolates were classified as diarrheagenic *E. coli* (DEC). This work highlights the potential transmission of antimicrobial-resistant bacteria through bivalves in the food chain. The ‘One Health’ approach is crucial for combating antimicrobial resistance, namely in edible freshwater species, emphasizing active surveillance to protect human, animal, and environmental health against the spread of antibiotic-resistant bacteria in aquatic environments.

## 1. Introduction

Bivalves are filter-feeding animals able to accumulate contaminants and microorganisms. Freshwater bivalves (FBs), such as mussels, are widespread in environments, like the Tua River, where they act as natural filters, concentrating particles, including bacteria [1]. This crucial ecological function not only makes them effective bioindicators of water quality, but also potential reservoirs for microbial pathogens [1,2]. Among the Portuguese species of freshwater bivalves, the focus of this study is centered on the autochthonous species, *Anodonta anatina*. This species can be found in Europe and Asia, from the Iberian Peninsula, in the south, to Scandinavia, in the north, and Russia, in the east. In terms of habitat selection and host fish utilization, *A. anatina* is considered a generalist species, as it colonizes lotic and lentic systems, including streams, large rivers, lakes, and reservoirs [3].

Antimicrobial resistance (AMR) refers to the absence or diminished effectiveness of antimicrobial agents in inhibiting bacterial growth and, in cases involving pathogenic microorganisms, this resistance can lead to treatment failure [4], being recognized as one of the ten major global public health concerns [5]. Indeed, AMR is responsible for numerous deaths annually worldwide, with a significant portion occurring within the European Union [6,7]. Antibiotic-resistant bacteria (ARB) can be acquired by humans through various means, including exposure in healthcare facilities, community settings, and contact with animals or contaminated environments [7,8,9].

*Escherichia coli* is an indicator of fecal contamination in food, marine, and freshwater environments, and has also been suggested as a possible indicator to assess the AMR status in environmental settings [10]. While most *E. coli* strains in the human gut are commensal, certain strains exhibit a pathogenic potential [11], which are responsible for causing diarrhea diseases and based on virulence traits can be grouped in different pathotypes: enteropathogenic (EPEC), shiga toxin-producing (STEC), enteroaggregative (EAEC), enterotoxigenic (ETEC), enteroinvasive (EIEC), and diffusely adherent *E. coli* (DAEC) [12]. In addition, there are also the extraintestinal pathogenic (ExPEC) strains that include neonatal meningitis-associated *E. coli* (NMEC), uropathogenic *E. coli* (UPEC), sepsis-causing (SPEC) and, avian pathogenic (APEC) [12,13].

Moreover, the use and misuse of antibiotics have led to the emergence of multidrug-resistant (MDR) *E. coli* strains, posing significant challenges for clinical treatment and public health management [14]. Integrating indicators of fecal contamination, such as MDR *E. coli*, into ecological assessments could enhance monitoring efforts, aligning with the ‘One Health’ concept, which emphasizes the interrelation of human, animal, and environmental health [15,16]. The presence of antibiotics in aquatic environments is an escalating concern due to their pervasive use in agriculture, human medicine, and industrial applications. These substances are often introduced into water bodies through agricultural runoff, sewage effluents, aquaculture, and industrial discharges, leading to the contamination of rivers, lakes, and streams [17,18,19,20,21]. Antibiotics can disrupt the microbial ecology of water bodies, impacting primary producers, such as algae [22,23], posing risks to higher trophic levels, including humans, who consume contaminated fish and shellfish [4,24]. The mobility of ARB through interconnected water systems facilitates the global dissemination of resistance, complicating international efforts to manage and contain antimicrobial resistance [10,25,26]. This contamination has far-reaching implications, affecting ecological health, water quality, and public health.

The Clermont phylogenetic method categorizes *E. coli* into distinct phylogroups, which are associated with varying pathogenic potentials and ecological niches [27,28]. Different phylogroups, such as B2 and D, are linked to specific pathogenic strains that cause serious infections, while others, like A and B1, are typically less harmful. This classification aids in identifying the sources and risks posed by different *E. coli* strains, which is essential for public health, epidemiology, and the development of targeted interventions [28].

Although bacteria serve as a feeding source for bivalves, they can also inhabit the body tissues of healthy individuals outside the gut. Mussels possess the ability to establish either mutually beneficial or antagonist symbiotic relations with bacteria [29,30]. Given the growing consumption of raw or undercooked foods, understanding the potential transmission of MDR *E. coli* through FBs is essential for mitigating public health risks [31]. The present study aims to evaluate the phylogenetic diversity, pathotypic characterization, and antimicrobial susceptibility of *E. coli* isolates from FBs sampled within the Tua River region in Portugal.

## 2. Materials and Methods

### 2.1. Freshwater Bivalve Collection

Fifteen *A. anatina* freshwater bivalves were collected from the Tua River basin (year 2022) at two different locations, location I (Chelas 41°30′45.82′′ N; 7°12′32.92′′ W: 7 FB) and location II (Barcel 41°24′18.69′′ N; 7°9′38.93′′ W: 8 FB), which are, respectively, upstream and downstream of Mirandela city (northeast of Portugal) [32]. The characterization of the study area and sampling processing are fully described in previous studies [2,32]. The freshwater bivalves were collected and maintained alive in a cooler with moist towels and transported to the Department of Veterinary Sciences, Antimicrobials, Biocides & Biofilms Unit (AB2Lab-DCV, CITAB), of the University of Trás-os-Montes and Alto Douro (UTAD), located in Vila Real, Portugal. The sampling of mussels was carried out with a permit granted by the Institute for the Conservation of Nature and Forestry (ICNF). No ethics committee approval was needed, and no animal experiments were performed in the scope of this research. 

### 2.2. Sample Processing, Isolation, and Identification of Bacteria

The fifteen FBs (*A. anatina*) were subjected to measurements of shell dimensions, followed by an aseptic opening to collect and weigh soft tissues. These tissues were then transferred to flasks containing Brain Heart Infusion (BHI) medium and incubated at 37 ± 1 °C for 24 h. Subsequently, the samples were inoculated on Chromocult^®^ Coliform Agar (CCA^®^) (Oxoid, Basingstoke, UK), a chromogenic medium, and the plates further incubated at 37 ± 1 °C for 24 h. Presumptive *E. coli* colonies were identified based on their characteristic blue/purple coloration on CCA^®^ according to the manufacturer’s guidelines. Confirmation of characteristic colonies was carried out using lactose fermentation on MacConkey agar plates and standard biochemical test-IMViC reactions (Indol, Methyl-red, Voges-Proskauer, and Citrate) were inoculated and incubated at 35–37 ± 1 °C for 24 h. Each bacterial isolate was assigned a specific code comprising letters (LI/LII) indicating the sampling location, Chelas (LI) or Barcel (LII), followed by an alphanumeric code representing the individual bivalve, and the designation “Ec” standing for *E. coli* followed by a number representing the strain (e.g., LIFB1Ec1), as can be observed in Table 1.

### 2.3. Antimicrobial Susceptibility Test

Susceptibility assays were performed using the agar disk diffusion method, following the Kirby–Bauer technique, in accordance with the guidelines provided by the European Committee on Antimicrobial Susceptibility Testing (EUCAST) [33], on Mueller–Hinton (MH) agar plates (OXOID, UK). A total of twenty-one antibiotics, covering seven antibiotic classes, was employed in the susceptibility assays: β-lactams (including penicillins: amoxicillin (AML, 10 μg), amoxicillin/clavulanic acid (AMC, 20/10 μg), piperacillin (PRL, 100 μg), and piperacillin/tazobactam (TZP, 100/10 μg); cephalosporins: cefoxitin (FOX, 30 μg), ceftazidime (CAZ, 30 μg), cefotaxime (CTX, 30 μg), and ceftriaxone (CRO, 30 μg); carbapenems: imipenem (IMP, 10 μg), meropenem (MEN, 10 μg), and ertapenem (ETP, 10 μg); and monobactam: aztreonam (ATM, 30 μg). Additionally, fluoroquinolones: ciprofloxacin (CIP, 5 μg); aminoglycosides: kanamycin (K, 30 μg), tobramycin (TOB, 10 μg), gentamicin (CN, 30 μg), and amikacin (AK, 30 μg); sulphonamides: sulfamethoxazole/trimethoprim (SXT, 23.75/1.25 μg); amphenicols: chloramphenicol (C, 30 μg); tetracyclines: tetracycline (TE, 30 μg); and fosfomycin (FOS, 50 μg) were included. Interpretation of the results was based on the breakpoints guidelines provided by the Clinical and Laboratory Standards Institute [34]. The bacteria were classified as susceptible (S), intermediate (I), or resistant (R). Reference strain *E. coli* ATCC 25922 was adopted as the control strain. Isolates exhibiting resistance to at least three different antimicrobial groups were categorized as multidrug-resistant (MDR) [35]. 

### 2.4. Phylogenetic Determination of E. coli Isolates

To determine the phylogenetic groups (A, B1, B2, D, E, F, and clade I) of *E. coli*, the multiplex PCR method described by Clermont et al. was employed [27]. This involved the amplification and sequencing of six conserved housekeeping genes (chuA, yjaA, Tsp.E4.C2, arpA, arpA (group E), and trpA (group C)). Specific primers for PCR amplification were synthesized by STAB-Vida (Caparica, Portugal), as listed in Table 2.

In brief, DNA extraction was performed using the GF-1 Bacterial DNA Extraction Kit (Vivantis, Shah Alam, Malaysia) following the manufacturer’s instructions. Subsequently, the concentration of DNA samples was quantified using a Biotek Powerwave XS2 Microplate Reader (Agilent Technologies, Winooski, VT, USA). by measuring absorbances at A260 nm and A280 nm, with sample purity assessed by the A260/A280 nm ratio. An evaluation of DNA integrity was performed through 0.6% agarose gel electrophoresis, dyed with 2 μL GreenSafe dye.

Each reaction was performed in a total volume of 20 µL. The reaction mixture comprised 3 µL of genomic DNA (30 ng/µL), 10× PCR buffer, 2 mM of each deoxyribonucleotide triphosphate (dNTP), 2 units of Taq polymerase (Bioron), 25 mM of MgCl_2_, 10 µL of specific forward and reverse primers (Table 2), and ddH_2_O to obtain the final volume.

The PCR reactions proceeded under the following conditions: initial denaturation at 95 ± 1 °C for 5 min, followed by 34 cycles consisting of denaturation at 94 ± 1 °C for 45 s, annealing at 57 ± 1 °C for 45 s (for group E) or 59 ± 1 °C for 45 s (for quadruplex and group C), extension at 72 ± 1 °C for 1 min, and a final extension step at 72 ± 1 °C for 5 min. The procedure was finalized by electrophoresis on 2% agarose gels in 1× Tris-borate-EDTA (TBE) buffer supplemented with GreenSafe DNA Gel Stain. The phylogenetic groups of each strain were determined after analyzing the electrophoresis gel, through the presence and/or absence of the genes represented in Table 2. 

### 2.5. Determination of E. coli Pathotypes

DNA extraction was performed using NZYTECH bacterial Cell Lysis Buffer (Ref. MB17801, NZYTECH, Coimbra, Portugal) with heat treatment at 95 °C for 15 min followed by centrifugation at 10,000 rpm for 3 min.

The identification of *E. coli* pathotypes (ETEC, EIEC, EAEC, and EHEC/STEC) was conducted through the real-time multiplex PCR technique using 20 µL of mixture (specific primers and probes for PCR amplification synthesized by Eurofins Genomics, Germany, ultra-pure water and mastermix with hot start temperature from NZYTECH, Portugal, ref. MB23003) and 5 µL of DNA template. Primers and probes were described by the EURL-VTEC_Method 02 for EHEC/VTEC [36], the EU-RL VTEC_Method_07 for EIEC [36], the EU-RL VTEC_Method_08 for ETEC [36], the EURL-VTEC_Method_05 for EAEC [36], and the ISO/TS 13136:2012 standard [37] and are defined in Table 3. Positive controls, including *E. coli* strains, LMV_E_37 (eae+; bfp+), LMV_E_38 (est+), LMV_E_39 (12 et+), LMV_E_40 (ipaH+), LMV_E_41 (aggr+; cvd432+), and O157:H7 (eae+; stx1+; stx2+), were used.

The thermoprofile used for the real-time PCR reaction was the initial denaturation step at 95 °C for 3 min, followed by 40 cycles consisting of denaturation at 95 °C for 15 s, annealing at 52 °C for 25 s, and extension at 72 °C for 30 s. 

## 3. Results

### 3.1. Bivalve Characterization

Despite being listed by the International Union for Conservation of Nature as Least Concern [38,39], *A. anatina* has suffered a strong decline in the last 20 years [40] and, as such, there was a rule to collect only 5% of the total number of individuals existing at the sampling sites. The characteristics of each bivalve are specified in Table 4. 

### 3.2. Antimicrobial Susceptibility Tests

**Figure 1:**
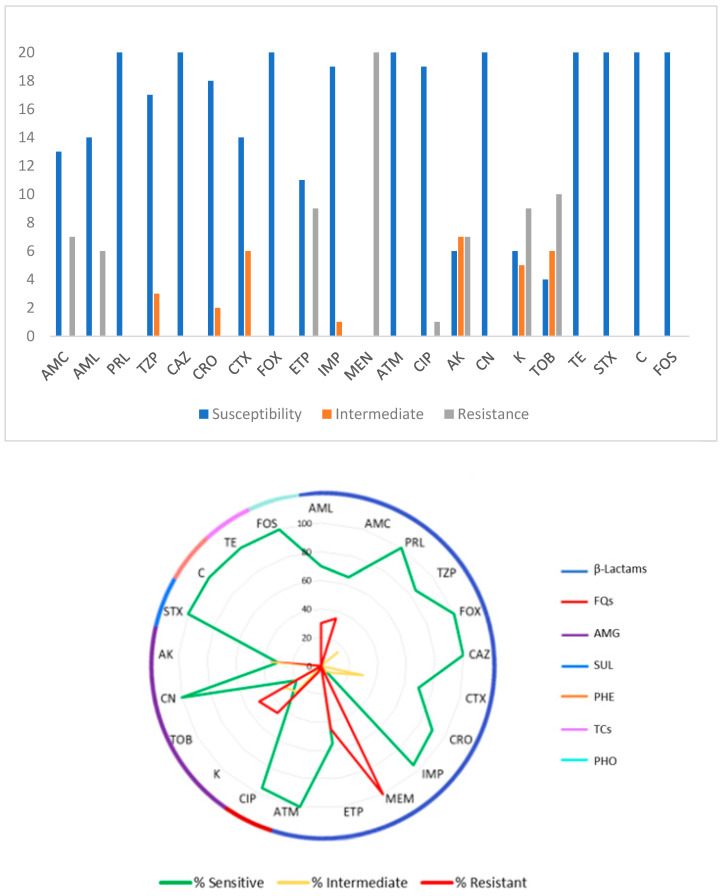
Susceptibility and resistance profiles of *E. coli* (*n* = 20) isolates to 21 antibiotics: AML—amoxicillin; AMC—amoxicillin/clavulanic acid; PRL—piperacillin; TZP—piperacillin/tazobactam; FOX—cefoxitin; CAZ—ceftazidime; CTX—cefotaxime; CRO—ceftriaxone; IMP—imipenem; MEM—meropenem; ETP—ertapenem; ATM—aztreonam; CIP—ciprofloxacin; AK—amikacin; CN—gentamicin; K—kanamycin; TOB—tobramycin; STX—sulfamethoxazole/trimethoprim; C—chloramphenicol; TE—tetracycline; FOS—fosfomycin.

Figure 1 presents an overview of the antimicrobial susceptibility of the twenty *E. coli* isolates. All the isolates showed susceptibility to PRL, FOX, CAZ, ATM, CN, STX, C, TE, and FOS. AK showed intermediate resistance for 35.0% (7 isolates) of the 20 isolates, CTX and TOB for 30.0% (6 isolates) each, K for 25.0% (5 isolates), TZP for 15.0% (3 isolates), CRO for 10.0% (2 isolates), and finally IMP for 5.0% (1 isolate). MEN was the antibiotic to which all isolates showed resistance (100%); in decreasing resistance order of the isolates: TOB with 50.0% (10 isolates), ETP and K with 45.0% (9 isolates), AMC and AK with 35.0% (7 isolates), AML with 30.0% (6 isolates), and CIP with 5.0% (1 isolate).

### 3.3. Multiresistant Isolates

The analysis of the resistance profiles for the twenty *E. coli* isolates showed that none of the isolates under study were susceptible to all the tested antimicrobial groups; however, 8 (40.0%) were resistant to two groups and 12 (60.0%) were MDR (resistant to three or more classes of antimicrobials). The largest number of isolates (10 isolates) exhibited resistance to three different antimicrobial classes; moreover, one isolate exhibited resistance to four classes and one to five classes. Table 5 reviews the multiple MDR patterns exhibited by the 20 isolates.

### 3.4. Phylogenetic Analysis and E. coli Pathotype Identification

**Figure 2:**
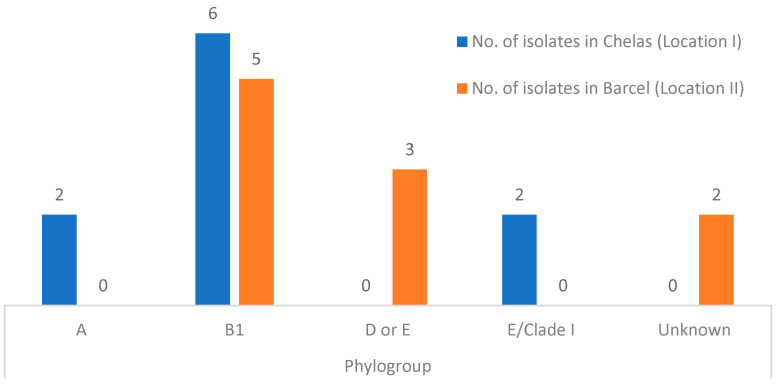
Phylogroup distribution detected in isolates from Chelas (LI) (blue) and Barcel (LII) (orange).

The classification of the *E. coli* isolates into the phylogroups was proposed by Clermont et al. [25]. Overall, 55% (11/20) belonged to phylogroup B1, 15% (3/20) to phylogroups D or E, 10% (2/20) to phylogroup A, 10% (2/20) to phylogroup E or Clade I, and for 10% (2/20) of the isolates it was not possible to identify to which phylogroup they belonged, thus being termed unknown. The results obtained are summarized in Figure 2.

The multiplex PCR analysis aimed at identifying *E. coli* pathotypes revealed that none of the twenty isolates harbored virulence genes associated with diarrheagenic *E. coli* strains. Hence, these isolates do not belong to pathogenic *E. coli* strains, indicating the predominantly commensal nature of the recovered strains in this study.

## 4. Discussion

The present study’s findings highlight the importance of freshwater bivalves as bioindicators for assessing contamination levels and associated risks to public health. *Escherichia coli*, a well-known indicator of fecal contamination in water sources, poses a significant global concern, particularly with the emergence of multidrug-resistant (MDR) strains [41]. Indeed, the present study aimed to investigate the phylogenetic diversity of *E. coli* isolated from freshwater bivalves (*Anodonta anatina*) and to characterize their phenotypes and antibiotic resistance profiles. There are no differences in the bivalves’ characteristics between locations, namely in length and weight. 

Out of the total isolates examined, twelve (60.0%) were classified as multidrug-resistant (MDR), demonstrating resistance to three or more antimicrobial classes. Consistent findings across multiple studies indicate a higher prevalence of antibiotic-resistant bacteria, including MDR strains, in organisms compared to water samples. Consequently, mollusks serve as a more reliable matrix for monitoring MDR bacteria, offering more robust assessment results than water samples. This highlights the importance of incorporating organism-based surveillance approaches into antimicrobial-resistance monitoring programs to better understand and address the spread of multidrug resistance in aquatic environments [42,43]. Noteworthy, all isolates exhibited resistance to meropenem, and the isolates from location I displayed resistance not only to meropenem, but also ertapenem, belonging to the carbapenem class, which are deemed last-resort antibiotics [44]. Additionally, amoxicillin and amoxicillin/clavulanic acid resistance were observed in Chelas (location I) in contrast to location II (Barcel), further distinguishing the resistance profiles between the two sites [2,32,45,46]. These variances maybe linked to the anthropogenic impact between the two locations. In location I (Chelas), the impacts mainly come from wastewater from hospitals, care facilities, and agriculture, whereas in location II (Barcel), they are primarily due to the agri-food industry present in the area.

In line with this result, previous research linked the presence of *E. coli* strains exhibiting higher levels of multidrug resistance due to anthropogenic influences. Varandas et al. stated that higher resistance rates were observed in locals with higher industrial activity, a larger population density, and pressures from livestock farming [41]. These findings align closely with the results of the present study, particularly in elucidating the marked differences in resistance profiles between location I and location II. This further emphasizes the intricate interplay between environmental factors, human activities, and the emergence and dissemination of antibiotic resistance in natural ecosystems. The emergence of multidrug-resistant *E. coli* strains is of paramount concern globally, given their rapid dissemination, as highlighted by the WHO [47]. These strains exhibit resistance not only to amoxicillin-based antibiotics, but also to fluoroquinolones, cephalosporins, and carbapenems, rendering conventional therapeutic options ineffective, as noted by a previous study [48]. Various studies have consistently shown that β-lactam antibiotics have been the preferred choice for treating infections caused by pathogenic strains of *E. coli*. However, the widespread use and sometimes inappropriate administration of these antibiotics have contributed to the emergence of β-lactamase-producing strains. This phenomenon emphasizes how closely patterns of antibiotic use and the emergence of antibiotic resistance in bacterial populations are. The development of innovative therapeutic approaches and prudent antibiotic prescribing practices are two crucial steps for halting the spread of resistance mechanisms and reducing the negative effects of antimicrobial resistance on public health [49,50]. The efficacy of β-lactam antibiotics is increasingly compromised by the rise in carbapenem-resistant Enterobacteriaceae, as highlighted by previous studies [43,51]. Alarmingly, existing protocols for assessing the hygiene and safety of harvested and distributed bivalves nationally and internationally do not incorporate evaluations for antimicrobial resistances that could be transmitted through these organisms, as noted by the WHO [47]. Addressing this gap in regulatory frameworks is imperative to safeguard public health and mitigate the spread of antibiotic resistance through foodborne pathways.

Concerning the phylogroups, the twenty isolates were allocated to one of the phylogroups delineated by Clermont et al. [27]. Each phylogroup plays a distinct ecological role, emphasizing the importance of categorizing *E. coli* strains into different groups to comprehend their pathogenicity, host interactions, and ecological impact on aquatic systems, as highlighted by other studies [41,51]. The author Giacometti et al. [51] noted that phylogroup B1 is among the most prevalent in mollusks, a finding consistent with the results of the present study. Furthermore, research by Bong et al. [52] demonstrated that multidrug-resistant isolates predominantly belong to phylogenetic group B1, further underlining the significance of phylogroup classification in understanding the dynamics of antimicrobial resistance in aquatic environments. Studies conducted on wastewater and surface water samples consistently indicate that most isolates belong to phylogroups A and B1, reflecting the prevalence of these groups in environments impacted by human activities, as demonstrated in various studies [53,54,55,56,57,58]. This observation aligns with research highlighting the robust survival capacity of strains in phylogroup B1 in aquatic environments [59,60]. The prevalence and resistance of phylogroup B1 in the studied bivalves may be attributed to its widespread distribution in this ecosystem. Notably, a significant proportion of the isolates characterized in this study belonged to phylogroups A and B1, suggesting a commensal origin, whereas *E. coli* strains classified as phylogroups B2 or D are typically associated with extraintestinal infections [50]. 

The real-time multiplex PCR analysis aimed at identifying *E. coli* pathotypes revealed that none of the isolates were associated with diarrheagenic *E. coli* groups. This finding underscores the primarily commensal nature of the isolated *E. coli* strains in this study, suggesting their potential role as indicators rather than direct pathogens in the aquatic environment. This distinction is crucial for understanding the ecological dynamics of *E. coli* populations and assessing the associated risks of antimicrobial-resistance transmission within aquatic ecosystems.

This study provides a comprehensive overview of freshwater bivalves as reservoirs of multidrug-resistant bacteria, shedding light on their potential implications for broader river ecosystems. Additionally, it underscores the emerging research domain of using freshwater bivalves as indicators for antimicrobial resistance, emphasizing the imperative need to further investigations to validate their effectiveness in this role. However, certain limitations are acknowledged, primarily stemming from the constrained collection of bivalves due to the endangered status of the species *A. anatina*. Moreover, the study underscores the importance of employing multidisciplinary methodologies to assess the ecological integrity of aquatic systems and advocates for the integration of microbiological analyses into ecosystem monitoring endeavors, guided by the ‘One Health’ concept. This holistic approach is essential for comprehensively understanding and addressing the complex dynamics of antimicrobial resistance in aquatic environments.

## 5. Conclusions

This work consisted in the study of *E. coli* strains recovered from freshwater bivalves in a river in Portugal. It revealed significant antimicrobial resistance, including to carbapenems, with variability in resistance rates between the two different sampling sites, linked to anthropogenic influences. The detection of carbapenem resistance is concerning due to potential food chain transmission. Most isolates belonged to commensal phylogroups, primarily B1, likely due to fecal contamination. These findings underline the importance of the One Health approach for monitoring and preventing the spread of antimicrobial resistance through aquatic environments, highlighting the need for further research and preventive measures to protect public and environmental health. Addressing this issue requires a comprehensive strategy that uses advancements in technology, regulatory frameworks, and public engagement to protect water quality and control the spread of antibiotic resistance.

## Figures and Tables

**Table 1:** Location and isolates’ identification.

Location	Isolates’ Identification
Chelas	LIFB1Ec1
	LIFB1Ec2
	LIFB2Ec1
	LIFB2Ec2
	LIFB4Ec1
	LIFB4Ec2
	LIFB5Ec1
	LIFB5Ec2
	LIFB6Ec1
	LIFB6Ec2
Barcel	LIIFB4Ec1
	LIIFB4Ec2
	LIIFB5Ec1
	LIIFB6Ec1
	LIIFB7Ec1
	LIIFB7Ec2
	LIIFB7Ec3
	LIIFB7Ec4
	LIIFB8Ec1
	LIIFB8Ec2

**Table 2:** Primers used by Clermont [27] in *E. coli* phylogenetic and amplicon size (bp).

Primer	Primer Sequences (5′-3′)	PCR Product (bp)
*chuA*	F: 5′-ATGGTACCGGACGAACCAAC-3′ R: 5′-TGCCGCCAGTACCAAAGACA-3′	288
*yjaA*	F: 5′-CAAACGTGAAGTGTCAGGAG-3′ R: 5′-AATGCGTTCCTCAACCTGTG-3′	211
Tsp.E4.C2	F: 5′-CACTATTCGTAAGGTCATCC-3′ R: 5′-AGTTTATCGCTGCGGGTCGC-3′	152
*arpA*	F: 5′-AACGCTATTCGCCAGCTTGC-3′ R: 5′-TCTCCCCATACCGTACGCTA-3′	400
*arp*A (group E)	F: 5′-GATTCCATCTTGTCAAAATATGCC-3′ R: 5′-GAAAAGAAAAAGAATTCCCAAGAG-3′	301
*trp*A (group C)	F: 5′-AGTTTTATGCCCAGTGCGAG-3′ R: 5′-TCTGCGCCGGTCACGCCC-3′	219

**Table 3:** Primers and probes used to determine *E. coli* pathotypes, according to the European Union Reference Laboratory for *E. coli*.

*E. coli* Pathotype	Gene	Primer Sequences (5′-3′)
EIEC	*ipah*	F: CCT TTT CCG CGT TCC TTG A R: CGG AAT CCG GAG GTA TTG C′ P: Cy5-CGC CTT TCC GAT ACC GTC TCT GCA BHQ2
ETEC	*lt*	F: TTCCCACCGGATCACCAA R: CAACCTTGTGGTGCATGATGA P: FAM-CTTGGAGAGAAGAACCCT BHQ1
ETEC	*sth*	F: GCTAAACCAGYAGRGTCTTCAAAA R: CCCGGTACARGCAGGATTACAACA P: HEX-TGGTCCTGAAAGCATGAA-BHQ1
ETEC	*stp*	F: TGAATCACTTGACTCTTCAAAA R: CCCCAGTTCARWGTRAGRTCMACRTC P: Cy5-TGAACAACACATTTTACTGCT BHQ2
STEC	*stx_1_*	F: TTTGTYACTGTSACAGCWGAAGCYTTACG R: CCCCAGTTCARWGTRAGRTCMACRTC P: FAM-CTGGATGATCTCAGTGGGCGTTCTTATGTAA-BHQ1
STEC	*stx_2_*	R: TTTGTYACTGTSACAGCWGAAGCYTTACG F: CCCCAGTTCARWGTRAGRTCMACRTC P:HEX-TCGTCAGGCACTGTCTGAAACTGCTCC-BHQ1
EAEC	*aggR*	R: CCTAAAGGATGCCCTGATGA′ F: GAATCGTCAGCATCAGCTACA P: FAM-CGGACAACTGCAAGCATCTA-BHQ1
EAEC	*aaiC*	R: CCTGATTTAGTTGATTCCCTACG F: CATTTCACGCTTTTTCAGGAAT P: HEX-CACATACAAGACCTTCTGGAGAA-BHQ1

**Table 4:** Characteristics of each bivalve collected.

	*A. anatina*	Length (mm)	Weight with Shell (g)	Weight of Soft Tissues (g)
Location I	FB1	65	19.869	12.148
	FB2	72	27.694	16.159
	FB3	83	30.522	17.736
	FB4	74	29.745	15.847
	FB5	81	33.917	19.064
	FB6	81	39.682	20.144
	FB7	73	37.190	20.120
Mean ± SD deviation		75.6 ± 6.40	31.23 ± 6.563	17.63 ± 2.866
Location II	FB1	69	23.502	13.369
	FB2	71	23.755	12.749
	FB3	75	34.533	18.081
	FB4	69	22.942	13.002
	FB5	72	24.785	11.492
	FB6	99	61.260	29.847
	FB7	98	56.498	29.250
	FB8	121	118.169	39.133
Mean ± SD deviation		86.4 ± 19.9	48.684 ± 34.406	21.936 ± 10.801

Comparations by Mann–Whitney test.

**Table 5:** Distribution of multidrug-resistant isolates of *E. coli* and antibiotic classes for which they exhibited resistance: PENs—penicillins; CEPs—cephalosporins; CARBs—carbapenems; FQs—fluoroquinolones; AMGs—aminoglycosides.

MDR Pattern	No. of Isolates in Chelas (Location I)	No. of Isolates in Barcel (Location II)	Total. No. of Isolates (%)
PEN-CEP-CARB-FQs-AMG	1	0	5
PEN-CEP-CARB-AMG	1	0	5
PEN-CARB-AMG	7	0	35
CEP-CARB-AMG	0	3	15
CARB-AMG	1	7	40

## Data Availability

Data are contained within the article.
